# Association of non-high-density lipoprotein cholesterol trajectories with the development of non-alcoholic fatty liver disease: an epidemiological and genome-wide association study

**DOI:** 10.1186/s12967-023-04291-4

**Published:** 2023-07-04

**Authors:** Jun-Hyuk Lee, Jiyeon Kim, Jung Oh Kim, Yu-Jin Kwon

**Affiliations:** 1 Department of Family Medicine, Nowon Eulji Medical Center, Eulji University School of Medicine, Seoul, 01830 Republic of Korea; 2 Department of Medicine, Hanyang University Graduate School of Medicine, Seoul, 04763 Republic of Korea; 3 Institute of Genetic Epidemiology, basgenbio Inc., 64, Keunumul-Ro, Mapo-Gu, Seoul, 04166 Republic of Korea; 4 Department of Family Medicine, Yongin Severance Hospital, Yonsei University College of Medicine, 363, Dongbaekjukjeon-daero, Giheung-gu, Yongin-si, Gyeonggi-do 16995 Republic of Korea

**Keywords:** Non-high-density lipoprotein cholesterol, Non-alcoholic fatty liver disease, Genome-wide association studies, Generic risk score, Trajectory model

## Abstract

**Background:**

Non-alcoholic fatty liver disease (NAFLD) shares common risk factors with cardiovascular diseases. Effects of longitudinal trends in non-high-density lipoprotein (non-HDL) cholesterol on NAFLD development are not understood. This study aimed to assess the relationship between non-HDL cholesterol trajectories and the incidence of NAFLD and to identify genetic differences contributing to NAFLD development between non-HDL cholesterol trajectory groups.

**Methods:**

We analyzed data from 2203 adults (aged 40–69 years) who participated in the Korean Genome and Epidemiology Study. During the 6-year exposure periods, participants were classified into an increasing non-HDL cholesterol trajectory group (*n* = 934) or a stable group (*n* = 1269). NAFLD was defined using a NAFLD-liver fat score > -0.640. Multiple Cox proportional hazard regression analysis estimated the hazard ratio (HR) and the 95% confidence interval (CI) for the incidence of NAFLD in the increasing group compared with the stable group.

**Results:**

A genome-wide association study identified significant single-nucleotide polymorphisms (SNPs) associated with NAFLD. During the median 7.8-year of event accrual period, 666 (30.2%) newly developed NAFLD cases were collected. Compared with the stable non-HDL group, the adjusted HR (95% CI) for the incidence of NAFLD in the increasing non-HDL cholesterol group was 1.46 (1.25–1.71). Although there were no significant SNPs, the polygenic risk score was highest in the increasing group, followed by the stable and control groups.

**Conclusion:**

Our study indicates that lifestyle or environmental factors have a greater effect size than genetic factors in NAFLD progression risk. Lifestyle modification could be an effective prevention strategy for NAFLD for people with elevated non-HDL cholesterol.

**Supplementary Information:**

The online version contains supplementary material available at 10.1186/s12967-023-04291-4.

## Introduction

Non-alcoholic fatty liver disease (NAFLD) shares a common pathophysiology with type 2 diabetes, obesity, dyslipidemia, and cardiovascular disease (CVD) [1]. Recently, a ‘multiple hit model’ has been accepted as a reasonable hypothesis for explaining the pathophysiology of NAFLD [2]. A sedentary lifestyle, poor eating habits, genetic factors, and epigenetic factors interact and synergistically modulate individual risk of NAFLD development.

Non-high-density lipoprotein (non-HDL) cholesterol, the result of subtracting high-density lipoprotein (HDL) cholesterol concentration from total serum cholesterol, is a strong predictor for CVD, which is the second most common cause of death in patients with NAFLD [3–5]. Although the influence of non-HDL cholesterol for CVD incidence has been established, there is a lack of data about the association between non-HDL cholesterol and NAFLD. A previous epidemiologic study revealed that non-HDL cholesterol level has a higher predictive power for the incidence of NAFLD than levels of total cholesterol, low-density lipoprotein (LDL) cholesterol, triglycerides, and HDL cholesterol [6]. In the aforementioned study, a total of 20.8% of people with a non-HDL cholesterol level between 130 and 160 mg/dL and 24.6% of those with a non-HDL cholesterol level > 160 mg/dL developed new-onset NAFLD whereas people with a non-HDL cholesterol level < 130 mg/dL did not develop NAFLD [6]. However, there is potential limitation in the previous study because only a spot-checked non-HDL cholesterol level was used, even though the non-HDL cholesterol level changes with time. Maintaining a lower non-HDL cholesterol level is suggested as the best strategy for the management of CVD [7], and thus, it should be a crucial issue whether changes in non-HDL cholesterol with time are significant to predict the incidence of NAFLD.

In the previous NAFLD GWAS study, genetic variants for pathogenesis and prognosis were discovered through various methods [8]. In particular, phospholipase domain-containing 3 (PNPLA3) [9, 10] and transmembrane 6 superfamily member 2 (TM6SF2) [10] are well known and replicated genetic markers that associated with hepatic fibrosis/cirrhosis [11, 12], hepatitis [13], and hepatocellular carcinoma [14, 15]. In addition, emerging GWAS studies on NAFLD are being established [16, 17].

As individuals advance into older age, typically around their 60 s and beyond, a decline in cholesterol levels is observed [18, 19]. The reasons for this decline are not entirely clear, changes in liver function and metabolism, which can affect cholesterol synthesis and regulation [18]. Interestingly, a previous study suggested that genetic factor can influence age related changes in total cholesterol and HDL cholesterol [19]. Although studies on the etiology, prognosis, and association of related diseases with NAFLD are actively being conducted, research on the aspect of preventive strategy is still insufficient. From this standpoint, it would be interesting to study the association of non-HDL cholesterol measurements, which are the important risk factors for CVD with NAFLD. Also, if there are genetic variations determining non-HDL cholesterol trajectories, early intervention to reduce non-HDL cholesterol levels should be applied as a preventive strategy for NAFLD. There is also a lack of evidence on the association of non-HDL cholesterol trajectories with the incidence of NAFLD. Therefore, this study aimed to verify the relationship between non-HDL cholesterol trajectories and the incidence of NAFLD. This study also focused on genetic differences contributing to the development of NAFLD in non-HDL cholesterol trajectory groups.

## Methods

### Study population

All analyzed data were derived from the Korean Genome and Epidemiology Study (KoGES)_Ansan_Ansung cohort. The KoGES_Ansan_Ansung cohort—a longitudinal, community-based cohort—has been conducted biennially by the Korea Centers for Disease Control and Prevention (KCDC) from the baseline survey (2001–2002) until the eighth follow up (2017–2018). A total of 10,030 participants, aged 40–69 years, were recruited in the cohort, which consists of 5,018 urban inhabitants (Ansan) and 5,012 rural inhabitants (Ansung) who lived in the areas at least 6 months. Information about personal medical histories, anthropometric measurements, and data from blood samples of each participant were collected at each visit.

Participants were followed from the date of the baseline survey until either the date at which the first NAFLD event was ascertained, the end date of the study, or the date of last informative contact. The time from the baseline survey to the third follow-up period was defined as the exposure period. The time from the third follow-up period to the eighth follow-up period was defined as the event accrual period. The incidence of NAFLD case was defined when a participant newly developed NAFLD during the event accrual period. The time interval from the third follow-up period to the time point at which the new-onset NAFLD event occurred was defined as the follow-up period.

**Fig. 1:**
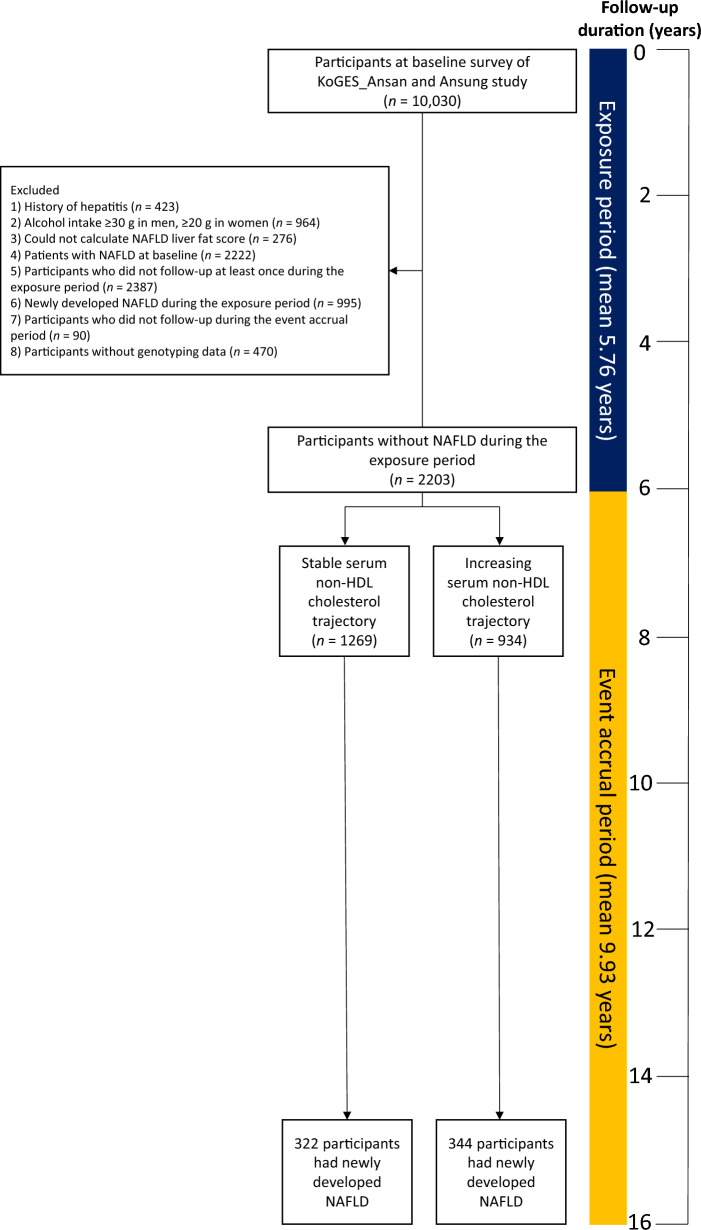
Flow chart of the study population

Figure 1 presents the flow of the study population selection. Among the initial 10,030 participants who took part in the baseline survey, we excluded the following groups: (1) individuals with a history of hepatitis (*n* = 423), (2) heavy drinkers who consumed at least 30 g/day of alcohol for men or 20 g/day for women (*n* = 964), (3) participants with insufficient data to calculate a NAFLD-liver fat score (*n* = 276), (4) individuals with NAFLD at the baseline survey (*n* = 2222), (5) participants who did not have at least one follow-up during the exposure period (*n* = 2387), (6) participants who developed NAFLD during the exposure period (*n* = 995), (7) participants who did not have follow-up data during the event accrual period (*n* = 90), and 8) those without genotyping data (*n* = 470). Finally, the analysis included a total of 2203 participants, comprising 1269 participants in the stable serum non-HDL cholesterol trajectory group and 934 participants in the increasing serum non-HDL cholesterol trajectory group. The institutional review board (IRB) of the KCDC reviewed and approved the KoGES_Ansan_Ansung cohort protocol. Each participant signed written informed consent. This study protocol conformed to the ethical guidelines of the 1964 Declaration of Helsinki and its later amendments. The IRB of Yongin Severace Hospital (IRB number; 9-2021-0081) approved this study.

### Data collection

Each participant’s height (cm) and weight (kg) were measured to the nearest 0.1 cm and 0.1 kg, respectively. Body mass index (BMI, kg/m^2^) was calculated. Waist circumference (WC, cm) was measured to the nearest 0.1 cm in the horizontal plane: midway between the lowest rib and the iliac crest. The average of the last two measured values were defined as the systolic blood pressure (SBP) and diastolic blood pressure (DBP); we also calculated the mean blood pressure (MBP).

Each participant was requested to respond to self-reported questionnaires regarding his/her diet, smoking status, alcohol drinking status, and physical activity. For the assessment of diet, a validated, 103-item semi-quantitative food frequency questionnaire was used. Total energy intake (kcal/day) was calculated. For smoking status, participants were classified as a never smoker, an ex-smoker, an intermittent smoker, or a daily smoker. The amount of alcohol intake (g/day) was calculated by multiplying the average amount of pure alcohol (10 g/per glass of drink), the number of glasses of alcoholic drinks consumed at a time (glasses/time), and the frequency of alcohol use (times/days). After excluding heavy drinkers, participants were divided into current drinkers or not. Physical activity of each participant was evaluated using an International Physical Activity Questionnaire. The metabolic equivalent of task (MET)-hours per day (MET-hr/day) was estimated and participants were classified into three categories according to their physical activity levels: low (< 7.5 MET-hr/day), moderate (7.5–30 MET-hr/day), or high (> 30 MET-hr/day).

After at least 8 h of fasting, blood samples of each participant were collected. Whole blood platelet count, fasting plasma glucose (FPG), concentrations of serum insulin, total cholesterol, triglyceride, HDL cholesterol, aspartate aminotransferase (AST), alanine aminotransferase (ALT), and C-reactive protein (CRP) were analyzed. Non-HDL cholesterol was calculated by subtracting serum HDL cholesterol level from serum total cholesterol level. In the case of serum triglyceride < 400 mg/dL, LDL cholesterol was calculated using the Friedewald formula.

We defined hypertension (HTN) as (1) a SBP ≥ 140 mmHg, (2) a DBP ≥ 90 mmHg, or (3) having treatment with anti-hypertensive medications [20]. Diabetes mellitus (DM) was defined as (1) a FPG ≥ 126 mg/dL, (2) a plasma glucose level ≥ 200 mg/dL at 2-h after the 75 g oral glucose tolerance test, (3) a glycosylated hemoglobin level ≥ 6.5%, (4) having treatment with anti-diabetic medications, or (5) having treatment with insulin therapy [21]. Dyslipidemia was defined as having serum total cholesterol concentration ≥ 240 mg/dL, LDL cholesterol concentration ≥ 160 mg/dL, HDL cholesterol concentration < 40 mg/dL, triglyceride concentration ≥ 200 mg/dL, or treatment with lipid-lowering medications [22].

### Serum non-HDL cholesterol trajectories

During the mean 5.76 years of the exposure period, temporal serum non-HDL cholesterol trends were determined by trajectory modeling with the concentration of serum non-HDL cholesterol at the baseline survey, first follow up, second follow up, and third follow up. We used group-based trajectory modeling to classify the trend of serum non-HDL cholesterol over time. This modeling assumes that participants are part of multiple trajectory groups capable of simultaneously estimating probabilities for multiple trajectories [23, 24]. According to these assumptions, the time-dependent covariates account for the variation in the mean trajectory within each group. The trajectories of serum non-HDL cholesterol of each group were classified using the r package ‘traj.’ In addition, the optimal number of non-HDL cholesterol trajectories of each group was evaluated using the r package ‘NbClust.’ Based on the trajectory modeling results, we categorized people into two groups, namely, (1) an increasing non-HDL cholesterol trajectory group and (2) a stable trajectory group (Additional file 1: Fig. S1).

### Assessment of NAFLD

To assess NAFLD status, we used a NAFLD-liver fat score. The formula for the NAFLD-liver fat score is as follows:

NAFLD-liver fat score = − 2.89 + 1.18 $\times$ metabolic syndrome (Yes: 1, No: 0) + 0.9 $\times$ DM (Yes: 1, No: 0) + 0.15 $\times$ insulin (µIU/mL) + 0.04 $\times$ AST (U/L)—0.94 $\times$ AST/ALT.

The presence of NAFLD was defined as a NAFLD-liver fat score greater than − 0.640 [25].

### Genotyping

Genomic DNA was extracted from the participants’ peripheral blood and genotyped using the Affymetrix Genome-Wide Human SNP Array 5.0  [26]. Single-nucleotide polymorphisms (SNPs) with minor allele frequencies (MAF) < 0.05, genotype calling rates < 95%, or deviated from the Hardy–Weinberg equilibrium (*p* < 1.0 $\times$ 10^–6^) were removed. Then, participants with inconsistent sex or calling rates at ~ 90% were excluded. Plink (v1.90) was used for quality control [27]. To impute the missing genotype data, the Beagle 5.0 software program was used [28]. Further details regarding the protocol have been described by Chung W et al. [28].

## Statistical analysis

### Epidemiologic data analysis

Based on the results of normality test, all data are presented as mean ± standard deviation or median (25th, 75th) for continuous variables or a number (percentage) for categorical variables. Student’s t-test or Mann–Whitney U test was used to compare the differences in continuous variables including age, BMI, WC, MBP, total energy intake, whole blood platelet counts and FPG, serum insulin, total cholesterol, triglyceride, HDL cholesterol, CRP, AST, and ALT levels between the two groups. A chi-squared test was used to compare differences in categorical variables, including smoking status, drinking status, physical activity, HTN, DM, and dyslipidemia, between groups.

The cumulative incidence rates of NAFLD during the event accrual period of the different trajectory groups are presented as Kaplan–Meier curves. The log-rank tests were used to determine whether distributions of the cumulative incidence rate of NAFLD differed between groups. Univariable and multivariable Cox proportional hazard regression analyses were performed to calculate the hazard ratio (HR) and 95% confidence interval (CI) for incidence of NAFLD. In Model 1, age and sex were included as confounding variables. In Model 2, age, sex, BMI, total energy intake, smoking status, drinking status, and physical activity were adjusted. In Model 3, variables used in Model 2, in addition to which HTN, DM, and serum CRP level were adjusted. In Model 4, serum ALT and triglyceride levels were further adjusted as a confounding variable, in addition to variables used in Model 3.

All statistical analyses were conducted using SAS version 9.4 (SAS Institute Inc., Cary, NC) and R software (version 4.1.1; R Foundation for Statistical Computing, Vienna, Austria). The significance level was set at *p* < 0.05.

### Genome-wide association analysis

To investigate NAFLD-related SNPs, we performed genome-wide association studies (GWAS) on the incidence of NAFLD phenotypes with 2203 participants. To identify NAFLD-related SNPs, single-variant association analysis was performed using a generalized mixed model that was implemented in the SAIGE R package (v0.45) [29] on genotype, and it imputed common variants of 2203 participants. The analysis was adjusted for age, sex, and 10 genotype principal components (PCs).

We hypothesized that participants in the non-HDL cholesterol increasing group had a higher risk to NAFLD incidence. To evaluate this hypothesis, the interaction polygenic risk scores (PRSs) [30] were applied to determine effects of SNPs on NAFLD phenotypes, which were estimated from participants in the increasing group by excluding the effects of SNPs from the stable group. PRSs were estimated using markers determined from interaction GWAS. Initially, we performed single-variant association analysis using a generalized mixed model for participants in the increasing group and the stable group separately. A model adjusted for age, sex, and 10 genotype PCs was constructed (Model 1). Subsequent models were constructed with adjustments for BMI, total energy intake, smoking status, drinking status, and physical activity (Model 2); HTN, DM, and serum CRP level (Model 3); and serum ALT level (Model 4). The covariates described above were added with covariates adjusted for each previous model. A total number of 4 models was utilized. Separately for each model described above, the interaction PRSs (Eq. 1) were calculated using the sum of differences between effect size of the increasing group and the stable group (Eq. 2), and the standard error for each SNPs were calculated according to Eq. 3  [30]. The *p* values were calculated using the standardized interaction PRS.

1 $$Interaction\, PRS = \mathop \sum \limits_{i}^{N} diff_{i} \times SNP_{i}$$

2 $$diff_{i} = {\text{beta}}_{{{\text{increasing}}}} - {\text{beta}}_{{{\text{stable}}}}$$

3 $$se\left( {diff_{i} } \right) = \sqrt {se\left( {{\text{beta}}_{{{\text{increasing}}}} } \right)^{2} + {\text{se}}\left( {{\text{beta}}_{{{\text{stable}}}} } \right)^{2} }$$

The parameter diff_i_ denotes the effect size of SNP i for the liver fat score-based NAFLD phenotype in the increasing group, excluding the effect size in the stable group; N denotes the total number of SNPs excluding those with a difference in minor allele frequency greater than 20%; and SNP_i_ denotes the number of i-th SNP’s effect allele.

### Comparing interaction PRS across trajectory groups

To investigate whether there were differences in the distribution of interaction PRS values derived from GWAS between the trajectory groups and the control group, we compared the interaction PRS for each group. To ensure a clear comparison between groups, we divided them into the control, stable, and increasing groups. For the control group, we first selected participants from the pool of 7827 people who were not included in the trajectory model and ensured that they had no history of NAFLD. After that, we randomly selected 2203 individuals to match the sample size used in the trajectory modeling, while the samples classified in the trajectory model were used as the stable group and increasing group.

The polygenic risk scores (PRS) between groups can be calculated by multiplying the dosage of SNPs present in each group with the effect size derived from interaction GWAS, as indicated in Eq. (1). By applying this calculation, we computed the PRS for the control group, stable group, and increasing group. Subsequently, these PRS values were normalized and presented in a forest plot.

## Results

### Baseline characteristics of the study population

Table 1 shows the baseline characteristics of the study population based on the non-HDL cholesterol trajectory groups (the increasing group and the stable group). For a total of 2203 participants, the mean age was 50.9 years, and the proportion of men was 40.4%. The increasing group exhibited higher mean values of BMI, WC, whole blood platelet count, FPG levels, serum total cholesterol, triglyceride, LDL cholesterol, and total energy intake compared to the stable group. Additionally, the proportion of participants with dyslipidemia was higher in the increasing group compared to the stable group. The mean serum HDL cholesterol level and the proportion of men, ex-smoker, intermittent smoker, daily drinker, current drinker, and participants with high level physical activity were lower in the increasing group than those in the stable group.

**Table 1:** Baseline characteristics of study population

	Total (*n* = 2203)	Serum non-HDL cholesterol trajectory groups		p^*^
		Stable (*n* = 1269)	Increasing (*n* = 934)	
Male sex, n (%)	891 (40.4%)	562 (44.29%)	329 (35.22%)	< 0.001
Age, years	50.9 ± 8.5	50.7 ± 8.7	51.0 ± 8.2	0.419
BMI, kg/m^2^	23.4 ± 2.6	23.2 ± 2.7	23.8 ± 2.5	< 0.001
WC, cm	78.5 ± 7.6	78.0 ± 7.6	79.3 ± 7.6	< 0.001
MBP, mmHg	92.0 ± 12.1	92.1 ± 12.2	91.9 ± 12.0	0.62
Smoking status, n (%)				0.030
Non-smoker	1537 (69.8%)	854 (67.3%)	683 (73.13%)	
Ex-smoker	277 (12.6%)	176 (13.87%)	101 (10.81%)	
Intermittent smoker	34 (1.5%)	21 (1.65%)	13 (1.39%)	
Daily smoker	355 (16.1%)	218 (17.18%)	137 (14.67%)	
Currently drinking, n (%)	953 (43.3%)	579 (45.63%)	374 (40.04%)	0.010
Physical activity, n (%)				< 0.001
Low	137 (6.2%)	75 (5.91%)	62 (6.64%)	
Moderate	1394 (63.3%)	754 (59.42%)	640 (68.52%)	
High	672 (30.5%)	440 (34.67%)	232 (24.84%)	
Platelet count, 10^9^/L	262.1 ± 59.8	257.1 ± 59.9	269.0 ± 59.1	< 0.001
FPG, mg/dL	81.7 ± 11.5	82.1 ± 12.0	81.1 ± 10.8	0.037
Insulin, μU/mL	6.4 ± 2.7	6.3 ± 2.7	6.5 ± 2.8	0.288
Total cholesterol, mg/dL	186.4 ± 32.8	176.5 ± 29.7	199.8 ± 32.0	< 0.001
Triglyceride, mg/dL	125.1 ± 59.0	115.3 ± 53.0	138.3 ± 63.9	< 0.001
HDL cholesterol, mg/dL	47.0 ± 10.0	47.5 ± 10.2	46.1 ± 9.7	0.001
LDL cholesterol, mg/dL	114.7 ± 29.7	106.1 ± 26.6	126.5 ± 29.5	< 0.001
eGFR, mL/min/1.73m^2^	93.4 ± 13.7	93.6 ± 13.9	93.1 ± 13.4	0.455
ALT, U/mL	21.2 ± 8.7	21.3 ± 8.9	21.1 ± 8.2	0.597
CRP, mg/dL	0.11 [0.05;0.19]	0.11 [0.04;0.19]	0.12 [0.05;0.20]	0.175
Total energy intake, kcal/day	1922.1 ± 656.5	1897.8 ± 619.7	1955.1 ± 702.5	0.047
HTN, n (%)	526 (23.9%)	311 (24.51%)	215 (23.02%)	0.448
DM, n (%)	37 (1.7%)	27 (2.13%)	10 (1.07%)	0.082
Dyslipidemia, n (%)	715 (32.5%)	335 (26.4%)	380 (40.69%)	< 0.001

BMI, body mass index; WC, waist circumference; MBP, mean blood pressure; FPG, fasting plasma glucose; HDL, high-density lipoprotein; LDL, low-density lipoprotein; eGFR, estimated glomerular filtration rate; ALT, alanine aminotransferase; CRP, C-reactive protein; HTN, hypertension, DM; diabetes mellitus

^*^p value for comparison of the baseline characteristics between participants in the stable trajectory and increasing trajectory groups; Significance was set at *p* < 0.05

### Longitudinal association of serum non-HDL cholesterol trajectories with the incidence of NAFLD during the event accrual period

**Fig. 2:**
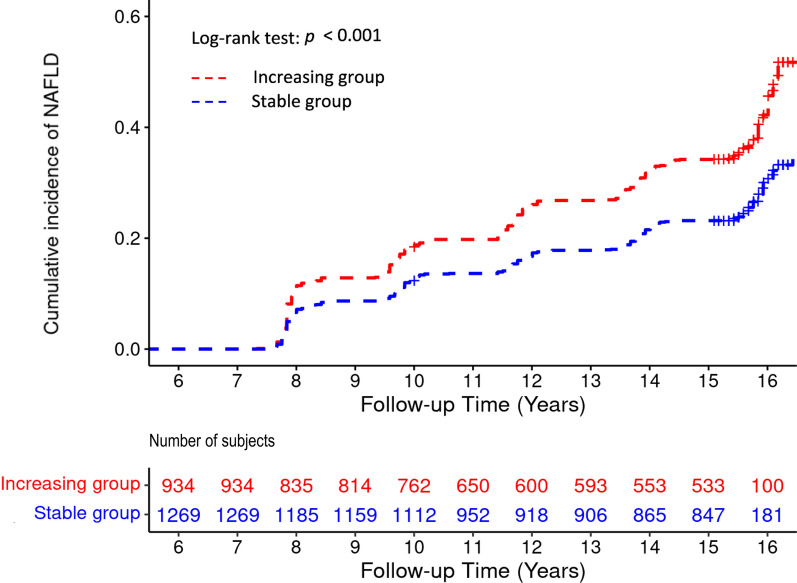
Cumulative incidence rate of NAFLD according to non-HDL cholesterol trajectory groups. NAFLD, non-alcoholic fatty liver disease; HDL, high-density lipoprotein

**Fig. 3:**
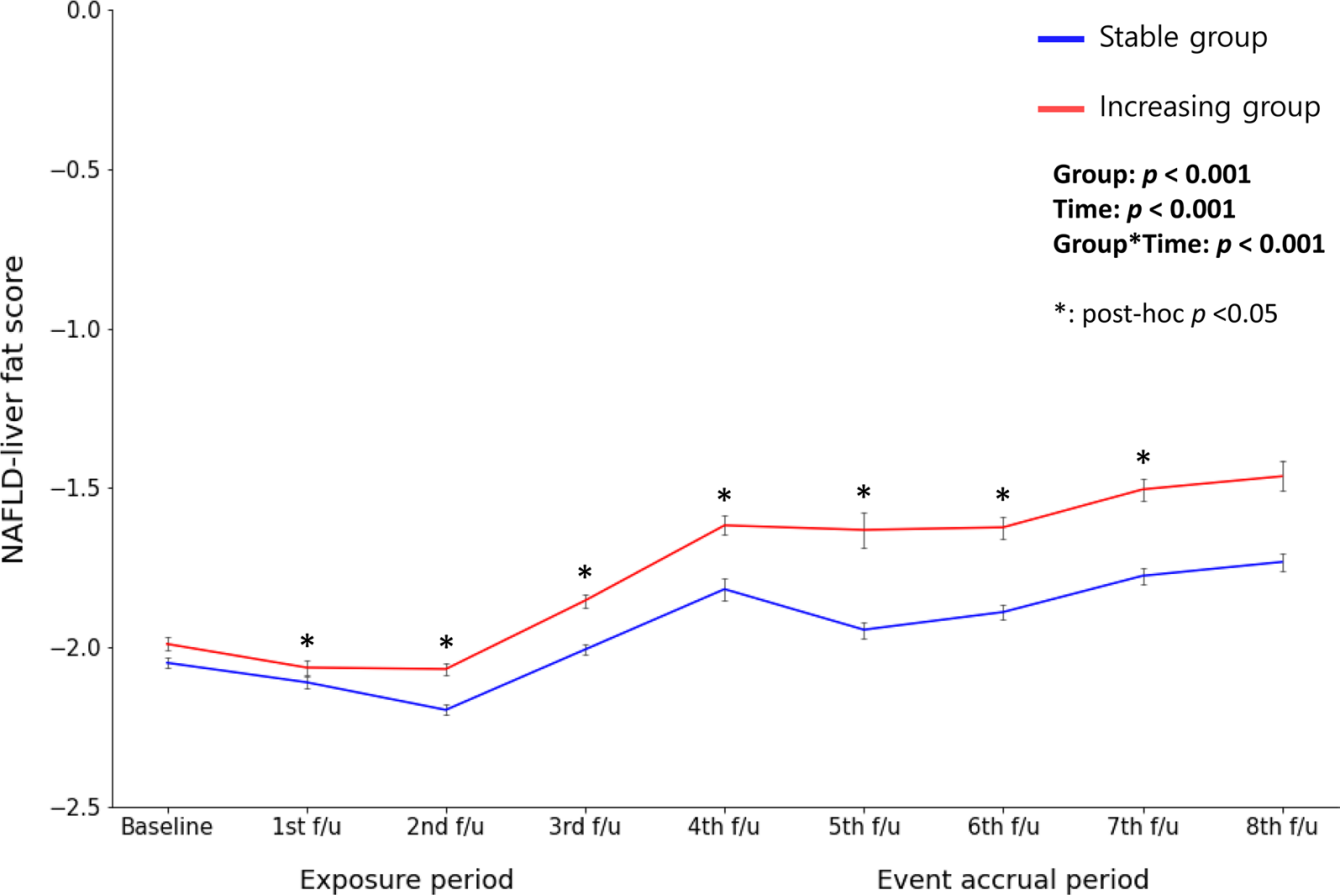
Longitudinal changes in NAFLD-liver fat score based on the non-HDL cholesterol trajectory groups. NAFLD, non-alcoholic fatty liver disease; HDL, high-density lipoprotein

During the mean 9.93 years of the event accrual period, there were 666 (30.2%) newly developed NAFLD cases. Incidence rates per 2 years of NAFLD ranged from 5.03 to 11.14 (Table 2). Figure 2 shows Kaplan–Meier curves for cumulative incidence rates of NAFLD of two different non-HDL cholesterol trajectory groups. During the event accrual period, the cumulative incidence rate of NAFLD in the increasing group was significantly higher than that in the stable group (log-rank test *p* < 0.001). Table 3 presents the HR with 95% CI for the incidence of NAFLD of two different non-HDL cholesterol trajectory groups using Cox proportional hazard regression analysis. Incidence rates per 1000 person-year in the stable group and the increasing group were 18.99 and 27.50, respectively. Compared with the stable group, the increasing group had significantly higher HR with 95% CI for the incidence of NAFLD (HR = 1.59, 95% CI 1.37–1.85, *p* < 0.001). The significant relationship remained in all adjusted models. In Model 4, the HR and 95% CI for the incidence of NAFLD of the increasing group compared with the stable group were 1.46 and 1.25–1.71, respectively (*p* < 0.001). Longitudinal changes in the NAFLD-liver fat scores of the increasing group and the stable group were analyzed using a linear mixed model, and the results are presented in Fig. 3. It was observed that the increasing group consistently had higher NAFLD-liver fat scores compared to the stable group during the follow-up periods. Furthermore, the analysis revealed a significant group-by-time interaction (*p* < 0.001), indicating that the rate of change in NAFLD-liver fat scores differed significantly between the two groups over time. It implies that the increasing group experienced a more pronounced increase in NAFLD-liver fat scores over time compared to the stable group. In the post-hoc analysis, the change in NAFLD-liver fat score from baseline to each follow-up period, except for the 8th follow-up, was found to be statistically significant between the two groups.

**Table 2:** Incidence of non-alcoholic fatty liver disease during follow-up

Period	Year range	Follow-up	Total (n)	Incidence cases (n)	Incidence rate per 2 years
Exposure period	2001–2008	Baseline to 3rd f/u	2203		
Event accrual period	2009–2018	4th f/u	2064	230	11.14
		5th f/u	1979	124	6.27
		6th f/u	1881	103	5.48
		7th f/u	1886	119	6.31
		8th f/u	1788	90	5.03

**Table 3:** Cox proportional hazard regression analysis for incidence of non-alcoholic fatty liver disease, in the different serum non-HDL cholesterol trajectory groups

	Stable group	Increasing group	
Total number, n	1269	934	
Incident NAFLD case, n	322	344	
Follow-up period, person-year	16958.1	12510.7	
Incidence rate per 1000 person-year	18.99	27.50	
	HR	HR (95% CI)	*p*
Unadjusted	1 (reference)	1.59 (1.37–1.85)	< 0.0001
Model 1	1 (reference)	1.60 (1.37–1.86)	< 0.0001
Model 2	1 (reference)	1.50 (1.29–1.75)	< 0.0001
Model 3	1 (reference)	1.54 (1.32–1.80)	< 0.0001
Model 4	1 (reference)	1.46 (1.25–1.71)	< 0.0001

Model 1: adjusted for age and sex

Model 2: adjusted for variables included in Model 1, as well as body mass index, smoking status, drinking status, physical activity, and total energy intake

Model 3: adjusted for variables included in Model 2, as well as hypertension, diabetes mellitus, and serum C-reactive protein level

Model 4: adjusted for variables included in Model 3, as well as serum alanine aminotransferase level and triglyceride level

NAFLD, non-alcoholic fatty liver disease; HR, hazard ratio; CI, confidence interval

### Genetic analysis results

**Fig. 4:**
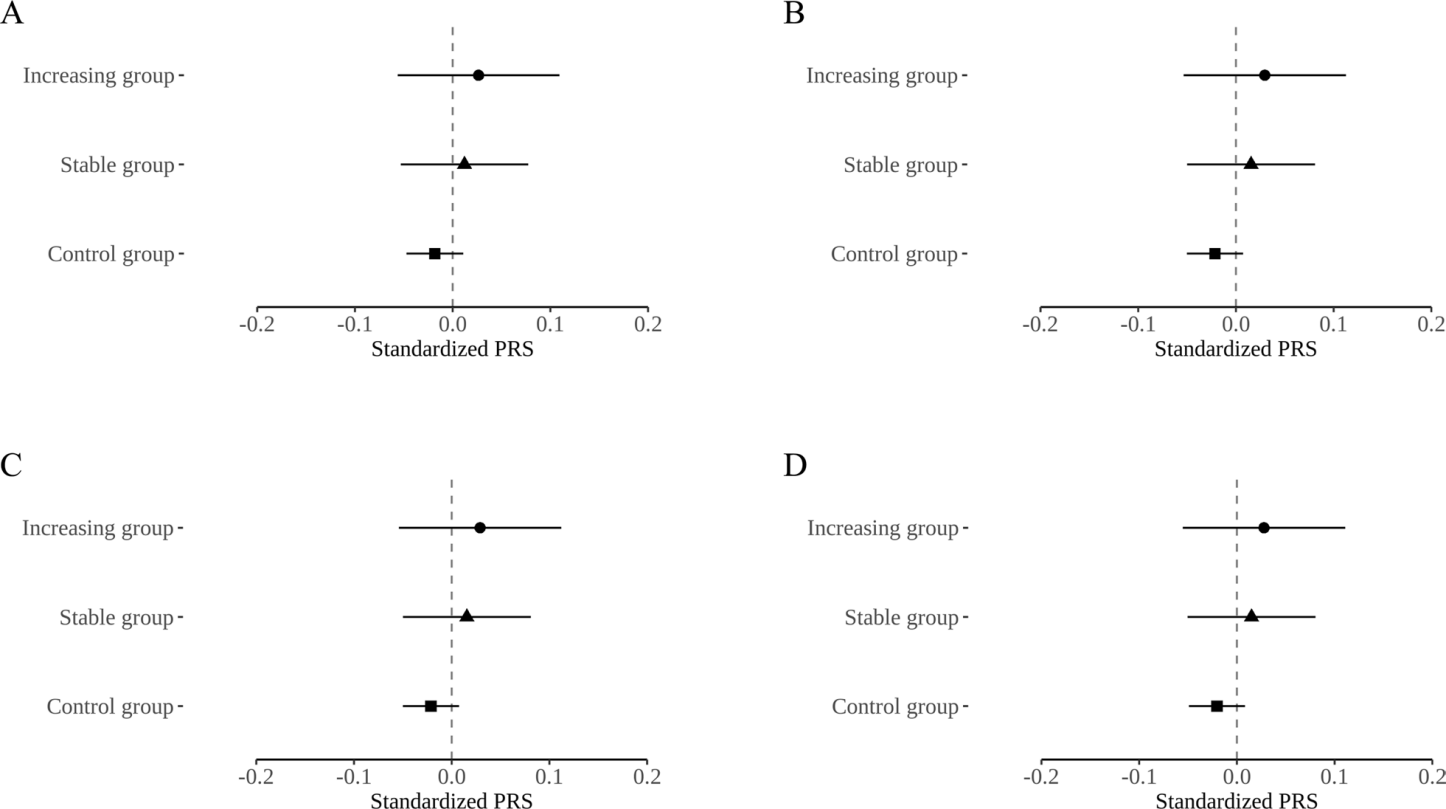
Forest plot comparing standardized PRS of NAFLD risk for the control, stable non-HDL cholesterol trajectory, and increasing non-HDL cholesterol trajectory groups in the interaction GWAS result. **A** Model 1, **B** Model 2, **C** Model 3, **D** Model 4. The models differ in the covariates used in the analysis. In Model 1, age and sex were included as confounding variables, PC1 ~ 10. In Model 2, age, sex, BMI, total energy intake, smoking status, drinking status, physical activity variables were used. In Model 3, the variables used in Model 2 plus HTN, DM, and serum CRP level were adjusted. In Model 4, the serum ALT level was further adjusted from Model 3. PRS, polygenic risk score; NAFLD, non-alcoholic fatty liver disease; HDL, high-density lipoprotein; GWAS, genome-wide association study; PC, principal component; BMI, body mass index; HTN, hypertension; DM, diabetes mellitus; CRP, C-reactive protein; ALT, alanine aminotransferase

Additional file 1: Fig. S2 shows the overall results of the GWAS. Although several loci were associated with NAFLD (*p* < 1.0 $\times$ 10^–5^), there were no SNPs reaching *p* ≤ 1.0 $\times$ 10^–8^. The quantile–quantile plot of the GWAS *p* values on NAFLD suggests no systemic over-dispersion of the association statistics (Additional file 1: Fig. S3). GWAS analysis with 4 models utilizing different covariates in each model confirmed that the effect estimates of SNPs were changed through the correction of potential confounding factors (Additional file 1: Fig. S4). Each Miami plot shows the significant SNPs (*p* ≤ 1.0 $\times$ 10^–5^; blue dotted line) for the incidence of NAFLD in the non-HDL cholesterol increasing (red) group and the stable (blue) group. Additionally, the results of GWAS on NAFLD incidence showed similar tendencies across all four models. The significantly associated SNPs were different between the increasing group and the stable group [weakly significant: *p* < -log10(1.0 $\times$ 10^–5^) or strongly significant: *p* < -log10(5.0 $\times$ 10^–8^)]. Additional file 1: Fig. S5 shows a Manhattan plot of the non-HDL cholesterol increasing group vs. the stable group interaction GWAS results. Although the interaction GWAS results did not show the genetic loci encoding the traditionally well-known GCKR, PNPLA3, and APOE genes in large cohort studies, the locus that formed the same cluster on the Manhattan plot for the four models showed that the loci included the neuregulin 1 (NRG1) and glypican-6 (GPC6) genes, i.e., a genetic locus well-known for its association with the pathogenesis of NAFLD. In the case of the NRG1 locus, the p-value of the analysis was < -log10 (1.0 × 10^–5^) without being affected by the model, and the GPC6 locus showed significance according to the adjusted confounding factor model. Figure 4 presents the forest plot of standardized PRS of NAFLD risk for the control, trajectory stable, and increasing trajectory groups. PRS was highest in the increasing group, followed by the stable group and the control group. Similar trends remained after adjusting for confounders.

## Discussion

In the epidemiologic data analysis, we found that the risk of the incidence of NAFLD in the non-HDL cholesterol group had increased by 54%, compared to the non-HDL cholesterol stable group, after adjusting for confounders. Moreover, changes in NAFLD-liver fat scores were significantly higher in the increasing group compared to the stable group from the 1st follow-up to the 7th follow-up period. However, at the 8th follow-up period, the difference in the changes in NAFLD-liver fat scores from baseline was not statistically significant. This finding suggests that, after a considerable number of NAFLD cases had occurred during the earlier follow-up period, it is possible that only individuals with a lower risk of NAFLD remained in the study population. There have been efforts to determine blood lipid profiles as predictors for NAFLD [6, 31, 32]. A clinical study in humans showed that impaired very-low-density lipoprotein (VLDL) secretion and deterioration of fatty acid oxidation induce serious lipid oxidation and DNA oxidative damage and contribute to the development of NAFLD [33]. Serum non-HDL cholesterol reflects pro-atherogenic lipoprotein containing apoprotein B better than VLDL, intermediate-density lipoprotein, and LDL [5]. In addition, a recent multi-dimensional study proved that administering statins is associated with a lower prevalence of non-alcoholic steatohepatitis and fibrosis through lowering both serum LDL and non-HDL cholesterol [34]. A randomized controlled trial also reported that ezetimibe combined with rosuvastatin significantly reduced liver fat in participants with NAFLD [35]. This evidence supports our results. Follow-up clinical trials are needed to confirm whether the reduction in serum non-HDL cholesterol level affects the reduction in intrahepatic steatosis directly or if it is simply a surrogate marker for reflecting intrahepatic steatosis.

In the genetic analysis, GWAS results for NAFLD did not identify significant SNPs (*p* < 1.0 $\times$ 10^–8^), and the weakly significant (*p* < 1.0 $\times$ 10^–5^) SNPs were also attenuated after adjusting for confounders unlike previous studies [9, 10, 16, 17], which might be evidence of a polygenic effect of SNPs on NAFLD phenotypes. One of the main reasons for the different results from previous studies can be an insufficient number of samples. Another reason may be that only newly developed NAFLD cases were included in the genetic analysis. Conversely, there are several possible reasons to support our findings. A study assessing the metabolic effects of the risk variants related to NAFLD (PNPLA3 and TM6SF2) did not show expected results [36]. The PNPLA3 variant rs738409-G is not associated with lipids, while TM6SF2 rs58542926-T is associated with lower concentrations of all VLDL, IDL, and LDL particles [36]. In the case of the stratified GWAS, according to the trajectory model presented, results suggested that the *p* value was met by clustering at the loci of the glucokinase regulator (GCKR), hepatocyte nuclear factor 1 homeobox A (HNF1A), and cholesteryl ester transfer protein. These loci, which have been reported to have an association with CVD in previous studies [37–39], have been reported to be associated with non-HDL cholesterol level. Among the loci encoded are the NRG1 and GPC6 genes, and a SNP in NRG1 locus was different between the increasing and the stable groups with weak significance independent of epidemiologic confounders. Neuregulins (NRGs) have gained attention as an essential family of signaling ligands regulating glucose and lipid homeostasis [40]. Previous studies suggest that the overexpression of the NRG family lowers blood glucose levels in obese mice and protects them from high-fat diet-induced hepatic steatosis [41, 42]. In particular, previous studies have shown that NRG1 promotes glucose uptake and mitochondrial oxidative metabolism to reduce blood sugar and weight gain, and more recent studies reported that NRG1 regulates the pathogenesis of NAFLD through ErbB3 signaling in hepatocytes. This suggests a sufficient probability that the marker of the locus is significant in the interaction GWAS comparing the non-HDL cholesterol-increasing group and the stable group in our results [43].

The significance of the loci, however, was attenuated in adjustment models, which may imply that the effect of lifestyle and environmental factors on serum HDL cholesterol metabolism outweigh that of expression of the GPC6 gene. In addition, Yoshida el al  [44]. identified the GPC6 locus through GWAS in patients with lean NAFLD compared with normal people. Due to the small sample size of the current study, we only analyzed data from the total population combining lean and overweight/obese. Therefore, the difference in the GPC6 locus between the increasing group and the stable group should be investigated in a future study with a larger sample size. Despite the small sample size of this study did not suggest a clear statistical power, and our results are sufficient to estimate the risk of the progression of NAFLD. Considering the significantly higher risk of the incidence of NAFLD of the increasing non-HDL cholesterol group compared with the stable group, lifestyles such as physical activity and eating habits or external environmental factors could have a greater effect size of the factors involved in NAFLD progression risk than genetic factors. Since NAFLD is a hepatic manifestation of metabolic abnormalities, it could be assumed that lifestyle and environmental factors may have played a greater role in the occurrence of NAFLD than genetic factors. Further studies with a larger sample size to investigate the interaction among multiple genetic, epigenetic, and environmental factors that determine an individual's susceptibility to NAFLD are needed.

There are possible explanations for the results in this study. First, serum non-HDL cholesterol would reflect impaired hepatic cholesterol metabolism, which increases the risk of intrahepatic steatosis. Abnormal hepatic cholesterol metabolism contributes to the development of atherosclerotic dyslipidemia  [45–47]. Both the influx of fatty acids to the liver and de novo lipogenesis increases intrahepatic triglyceride levels, which results in the increased oxidation of fat in the liver and an increase in the export of VLDL cholesterol from the liver to the blood [1, 48]. Moreover, there is a lower capacity for cholesterol efflux in patients with NAFLD compared to people without NAFLD [49]. The altered HDL cholesterol-mediated efflux of cholesterol and plasma loading capacities are found in patients with metabolically-driven NAFLD but not in patients with genetically-driven NAFLD carrying a M148M PNPLA3 genotype [50]. Second, dyslipidemia can induce hepatic insulin resistance through an increase in diacylglycerol and ceramide levels in the liver [51]. In this regard, a vicious cycle may exist in the elevated blood lipid profile and the development and progression of hepatic steatosis.

There are several limitations in this study. First, since data regarding imaging tools, such as abdominal ultrasonography, abdominal computed tomography, or transient elastography, was unavailable, NAFLD was determined using a surrogate marker, namely, a NAFLD-liver fat score. Second, there is the possibility of selection bias because we excluded people who had baseline NAFLD and newly developed NAFLD during the exposure period. Therefore, our results would not reflect those who are at a high risk for the incidence of NAFLD. Third, we only included middle-aged and older Korean adults, and thus, our results cannot be generalized to other ethnic populations. Finally, although our focus has been on the dynamic factor of non-HDL cholesterol, we acknowledge the importance of considering the impact of changing confounding variables, such as lifestyle factors and metabolic factors, on the incidence of NAFLD. Therefore, in future research, it is essential to take into account the transition status of these factors to accurately assess the risk of NAFLD occurrence. Despite these limitations, this is the first study to examine the association between the non-HDL trajectory group and NAFLD via a longitudinal prospective study. Additionally, for the first time, we estimated the risk factors of NAFLD considering genetic factors.

Increasing serum non-HDL cholesterol is a risk factor for the incidence of NAFLD. Although there were no significant SNPs in this GWAS study, PRS was the highest in the increasing serum non-HDL cholesterol group, followed by the stable serum non-HDL cholesterol and control groups. Our findings suggest that serum non-HDL cholesterol management could be a preventive strategy for NAFLD and CVD. Additional validation studies are warranted to investigate the effects of the risk factors, including the identified genetic factors for NAFLD development.

## Supplementary Information

**Additional file 1: Figure S1.** Trajectory modeling with non-HDL cholesterol using the latent class linear mixed model. Red line: non-HDL cholesterol increasing group, blue line: non-HDL cholesterol stable group. HDL, high-density lipoprotein. **Figure S2.** Manhattan Plot of the NAFLD-control analysis based on liver fat score. The P values are represented in genomic order by chromosome and position on the chromosome (x-axis). The value on the y-axis represents the −log10 of the p-value (equivalent to the number of zeros after the decimal point plus one). The blue dotted line indicates p-values ≤ 1.0 x 10^-5^. There are no SNPs reaching p-values ≤ 1.0 x 10^-8^. NAFLD, non-alcoholic fatty liver disease; SNP, single nucleotide polymorphism. **Figure S3.** Quantile-quantile plot of the GWAS p-values for NAFLD. The x-axis and y-axis represent the expected p-values and the observed p-values, respectively. The red line indicates observed p-values are equal to expected p-values. GWAS, genome-wide association study; NAFLD, non-alcoholic fatty liver disease. **Figure S4.** Miami Plot of the NAFLD-control analysis stratified on the trajectory model, the increased group, and the stable group for cholesterol excluding HDL cholesterol. (A) Miami plot of the GWAS result. (B) Miami plot of the Model 2 GWAS result. (C) Miami plot of the Model 3 GWAS result. (D) Miami plot of the Model 4 GWAS result. Each Miami plot shows results for NAFLD status in stable group (blue) and the increasing group (red). The models differ in terms of the covariates used for the analysis. In Model 1, age and sex were included as confounding variables, PC1~10. In Model 2, age, sex, BMI, total energy intake, smoking status, drinking status, and physical activity variables were used. In Model 3, the variables used in Model 2 were included, in addition to which HTN, DM, and serum CRP level were adjusted. In Model 4, the serum ALT level was further adjusted from Model 3. NAFLD, non-alcoholic fatty liver disease; HDL, high-density lipoprotein; GWAS, genome-wide association study; BMI, body mass index; HTN, hypertension; DM, diabetes mellitus; CRP, C-reactive protein; ALT, alanine aminotransferase. **Figure S5.** Manhattan plot of the increasing group vs. the stable group interaction GWAS results: (A) Model 1, (B) Model 2, (C) Model 3, and (D) Model 4. The models differ in the covariates used in the analysis. In Model 1, age and sex were included as confounding variables, PC1~10. In Model 2, age, sex, BMI, total energy intake, smoking status, drinking status, physical activity variables were used. In Model 3, the variables used in Model 2 were included, in addition to which HTN, DM, and serum CRP level were adjusted. In Model 4, the serum ALT level was further adjusted from Model 3. GWAS, genome-wide association study; PC, principal component; BMI, body mass index; HTN, hypertension; DM, diabetes mellitus; CRP, C-reactive protein; ALT, alanine aminotransferase.

## Data Availability

The dataset used in this study can be provided after review and evaluation of the research. Plan by the Korea Centers for Disease Control and Prevention. (http://www.cdc.go.kr/CDC/eng/main.jsp).
