# Supplementary material for: Association of non-high-density lipoprotein cholesterol trajectories with the development of non-alcoholic fatty liver disease: an epidemiological and genome-wide association study
Source: J Transl Med. 2023 Jul 4;21:435. doi: 10.1186/s12967-023-04291-4 (PMC10318766; doi:10.1186/s12967-023-04291-4)
Supplement: Supplementary file 1 — Additional file 1: Figure S1. Trajectory modeling with non-HDL cholesterol using the latent class linear mixed model. Red line: non-HDL cholesterol increasing group, blue line: non-HDL cholesterol stable group. HDL, high-density lipoprotein. Figure S2. Manhattan Plot of the NAFLD-control analysis based on liver fat score. The P values are represented in genomic order by chromosome and position on the chromosome (x-axis). The value on the y-axis represents the −log10 of the p-value (equivalent to the number of zeros after the decimal point plus one). The blue dotted line indicates p-values ≤ 1.0 x 10^-5^. There are no SNPs reaching p-values ≤ 1.0 x 10^-8^. NAFLD, non-alcoholic fatty liver disease; SNP, single nucleotide polymorphism. Figure S3. Quantile-quantile plot of the GWAS p-values for NAFLD. The x-axis and y-axis represent the expected p-values and the observed p-values, respectively. The red line indicates observed p-values are equal to expected p-values. GWAS, genome-wide association study; NAFLD, non-alcoholic fatty liver disease. Figure S4. Miami Plot of the NAFLD-control analysis stratified on the trajectory model, the increased group, and the stable group for cholesterol excluding HDL cholesterol. (A) Miami plot of the GWAS result. (B) Miami plot of the Model 2 GWAS result. (C) Miami plot of the Model 3 GWAS result. (D) Miami plot of the Model 4 GWAS result. Each Miami plot shows results for NAFLD status in stable group (blue) and the increasing group (red). The models differ in terms of the covariates used for the analysis. In Model 1, age and sex were included as confounding variables, PC1~10. In Model 2, age, sex, BMI, total energy intake, smoking status, drinking status, and physical activity variables were used. In Model 3, the variables used in Model 2 were included, in addition to which HTN, DM, and serum CRP level were adjusted. In Model 4, the serum ALT level was further adjusted from Model 3. NAFLD, non-alcoholic fatty liver disease; HDL, high-dens [file 12967_2023_4291_MOESM1_ESM.docx]

**Supplementary materials for:**

Association of non-high-density lipoprotein cholesterol trajectories with the development of non-alcoholic fatty liver disease: an epidemiological and genome-wide association study

Jun-Hyuk Lee MD^1,2†^, Jiyeon Kim^3†^, Jung Oh Kim PhD^3^, Yu-Jin Kwon MD, PhD^4^*,

^1^Department of Family Medicine, Nowon Eulji Medical Center, Eulji University School of Medicine, Seoul 01830, Republic of Korea

^2^Department of Medicine, Hanyang University Graduate School of Medicine, Seoul 04763, Republic of Korea

^3^Basbio

^4^Department of Family Medicine, Yongin Severance Hospital, Yonsei University College of Medicine, Yongin, 16995, Republic of Korea

^†^These authors are co-first authors who equally contribute to this work.

***Corresponding authors:**

Yu-Jin Kwon, MD, PhD

Assistant Professor, Department of Family Medicine

Yonsei University College of Medicine, Yongin Severance Hospital

363, Dongbaekjukjeon-daero, Giheung-gu, Yongin-si, Gyeonggi-do-16995, Republic of Korea;

Tel: +82 31 5189 8777; Fax: +82 3462 8209; Mobile: +82 10 4180 7414; Email: [digda3@yuhs.ac](mailto:digda3@yuhs.ac)

Jung Oh Kim, PhD

Principal Researcher,

Basgenebio, 64, Keunumul-ro, Mapo-gu, Seoul, Republic of Korea, 04166

Mobile: +82 10 8768 8505 Fax: 070 8740 8888; Email: [kjo@basgenbio.com](mailto:kjo@basgenbio.com)

**Supplementary Figure 1.** Trajectory modeling with non-HDL cholesterol using the latent class linear mixed model. Red line: increasing non-HDL cholesterol trajectory group, blue line: stable non-HDL cholesterol trajectory group.


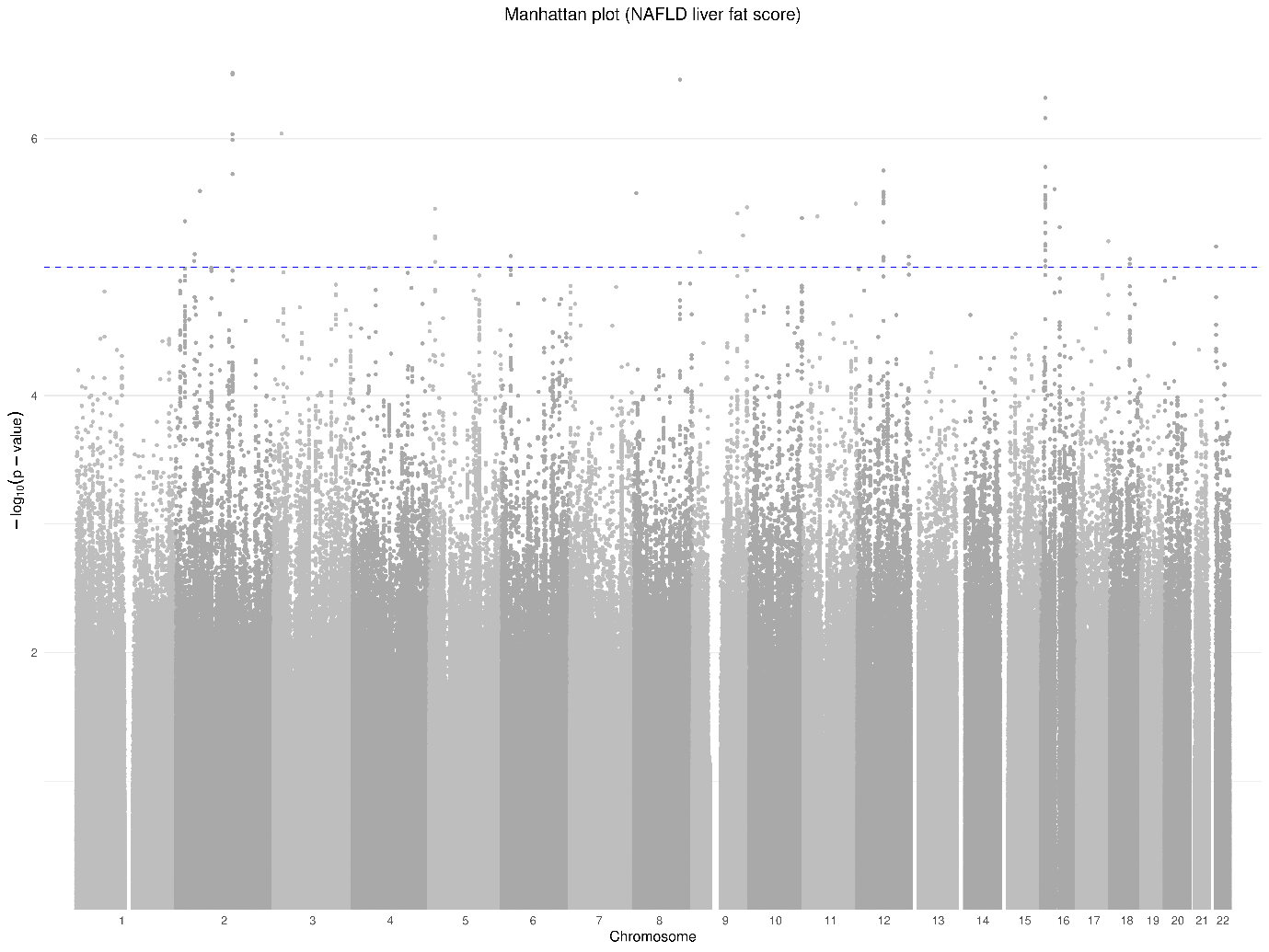


**Supplementary Figure 2.** Manhattan Plot of the NAFLD-control analysis based on liver fat score. The P values are represented in genomic order by chromosome and position on the chromosome (x-axis). The value on the y-axis represents the −log10 of the P value (equivalent to the number of zeros after the decimal point plus one). The blue dotted line indicates p-values ≤ 1.0 x 10^-5^. There are no SNPs reaching P ≤ 1.0 x 10^-8^.


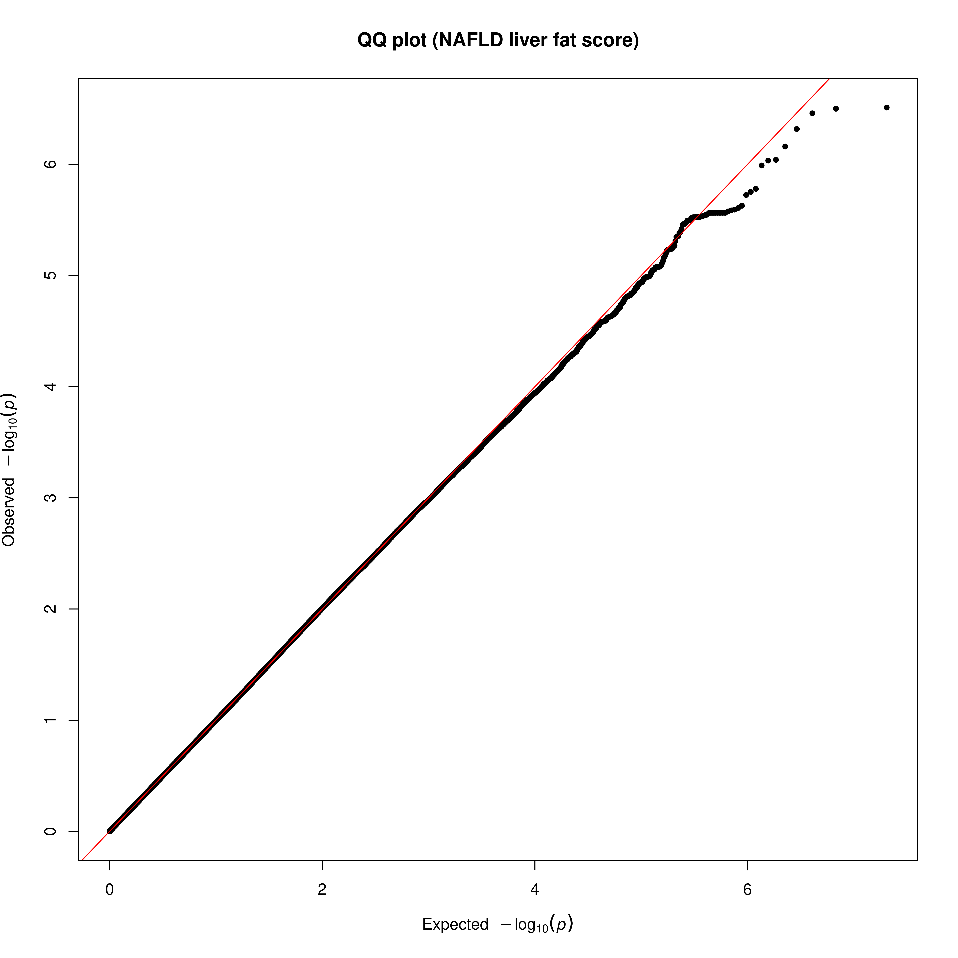


**Supplementary Figure 3.** Quantile-quantile plot of the GWAS p-values for NAFLD. The x-axis and y-axis represent the expected p-values and the observed p-values, respectively. The red line indicates observed p-values are equal to expected p-values.


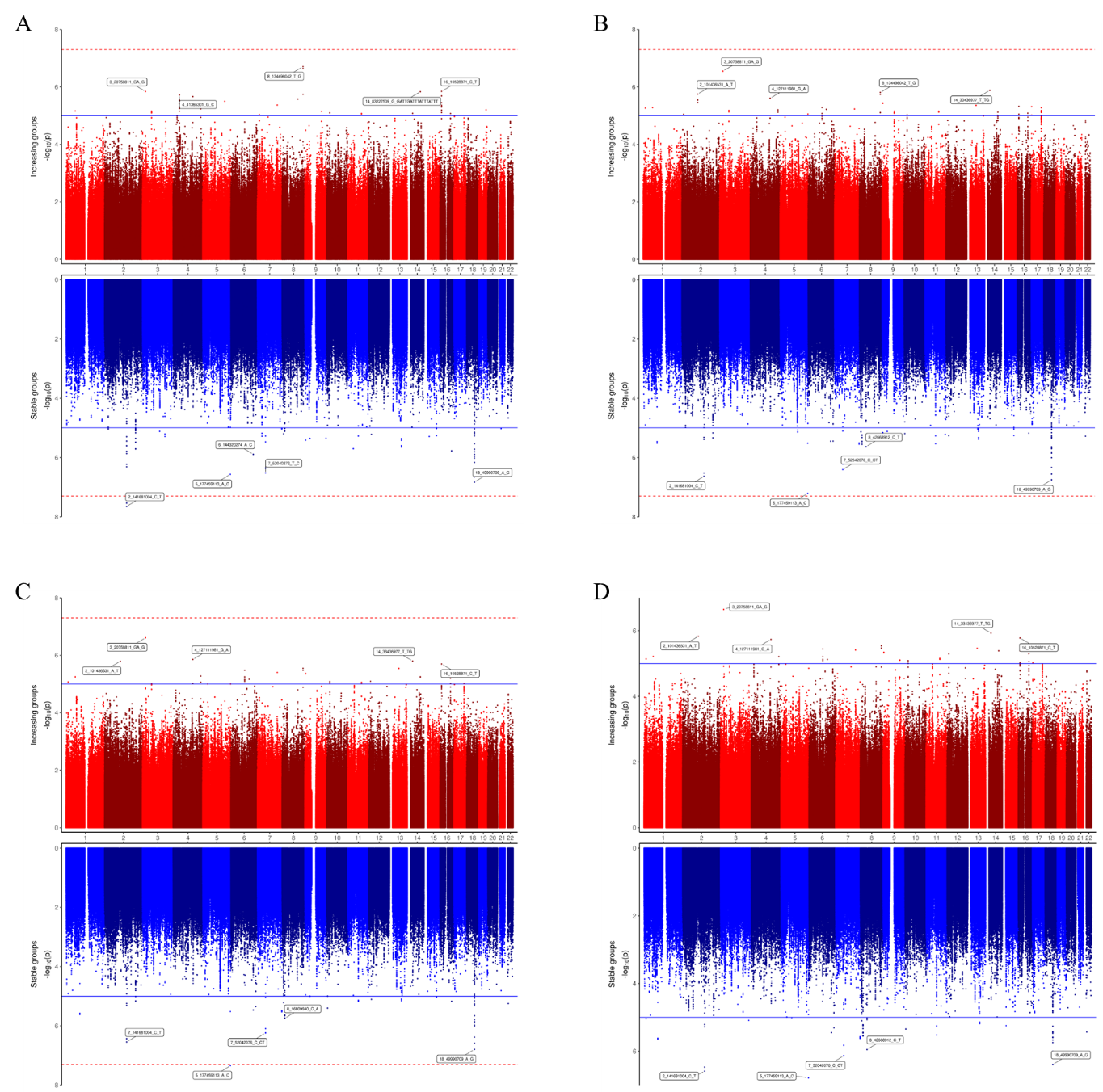


**Supplementary Figure 4.** Miami Plot of the NAFLD-control analysis stratified on the trajectory model, the increased group, and the stable group for cholesterol excluding HDL. (A) Miami plot of the Model 1 GWAS result. (B) Miami plot of the Model 2 GWAS result. (C) Miami plot of the Model 3 GWAS result. (D) Miami plot of the Model 4 GWAS result. Each Miami plot shows results for NAFLD status in stable group (blue) and the increasing group (red). The models differ in terms of the covariates used for the analysis. In Model 1, age and sex were included as confounding variables, PC1~10. In Model 2, age, sex, BMI, total energy intake, smoking status, drinking status, and physical activity variables were used. In Model 3, the variables used in Model 2 were included, in addition to which HTN, DM, and serum CRP level were adjusted. In Model 4, the serum ALT level was further adjusted from Model 3.


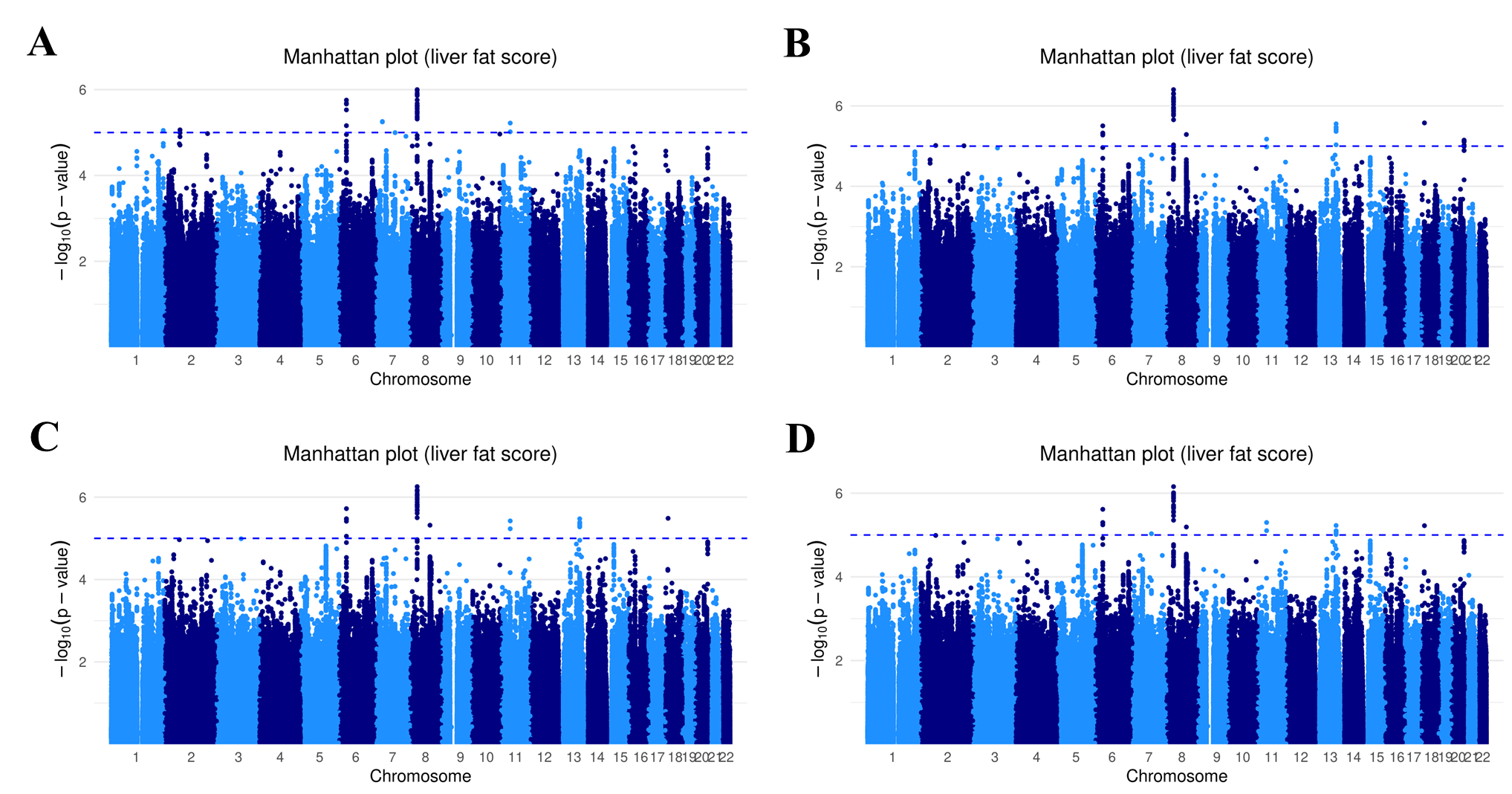


**Supplementary Figure 5.** Manhattan plot of the increasing group vs. the stable group interaction GWAS results: Models (A) 1, (B) 2, (C) 3, and (D) 4. The models differ in the covariates used in the analysis. In Model 1, age and sex were included as confounding variables, PC1~10. In Model 2, age, sex, BMI, total energy intake, smoking status, drinking status, physical activity variables were used. In Model 3, the variables used in Model 2 were included, in addition to which HTN, DM, and serum CRP level were adjusted. In Model 4, the serum ALT level was further adjusted from Model 3.
